# Cyanotoxins Occurrence in Portugal: A New Report on Their Recent Multiplication

**DOI:** 10.3390/toxins12030154

**Published:** 2020-02-29

**Authors:** Cristiana Moreira, Cidália Gomes, Vitor Vasconcelos, Agostinho Antunes

**Affiliations:** 1 CIIMAR/CIMAR, Interdisciplinary Centre of Marine and Environmental Research, University of Porto, Terminal de Cruzeiros do Porto de Leixões, Av. General Norton de Matos S/N, 4050-208 Porto, Portugal; cmoreira@ciimar.up.pt (C.M.); g.cidalia@gmail.com (C.G.); vmvascon@fc.up.pt (V.V.); 2 Department of Biology, Faculty of Sciences, University of Porto, Rua do Campo Alegre, 4169-007 Porto, Portugal

**Keywords:** Microcystins, Cylindrospermopsins, Anatoxin-a, Saxitoxins, PCR, ELISA, Risk assessment

## Abstract

Historical reports show that in Portugal, cyanotoxins reports were mainly in the Center (cylindrospermopsins) and South (cylindrospermopsins, saxitoxins) regions of the country apart from the well distributed microcystins. Therefore, in our study, seven freshwater ecosystems located in the North and Center Regions of Portugal were screened between April and September of 2017 for the main cyanotoxins (microcystins, cylindrospermopsins, anatoxin-a, and saxitoxins) by a two methods approach that combined the application of molecular (PCR) and immunological (ELISA) assays. Results from our survey reveal that both methods revealed the presence of all main cyanotoxins. ELISA results showed that 48% of the samples were above (1.6–18.8 μg/L) the guideline value established for microcystins (1 μg/L), while in the remaining cyanotoxins, 33% of the samples were above (1.1–6.8 μg/L) the guideline value established for anatoxin–a (1 μg/L). Further, for saxitoxins, only one sample gave a value above (4.3 μg/L) the guideline (3 μg/L) and this corresponded to a North Region ecosystem. In the cytotoxin cylindrospermopsins, none of the samples were above the guideline established value (1 μg/L). This study will improve the risk assessment strategy in Portugal, as well as advance water quality and water management.

## 1. Introduction

Cyanobacteria represent a great public health concern through the formation of dense agglomerations on aquatic ecosystems that consequently produce and release harmful cyanotoxins. Another important factor in cyanobacteria damage is the impairment of water quality through the release of compounds that interfere with the odor and taste of freshwater and the production of surface scums prior to bloom events. With this, the constant vigilance of ecosystems prior, during, or post bloom events becomes mandatory to determine the level of cyanotoxicity that is initially present or produced during or after a bloom. Cyanotoxins are, therefore, natural molecules that are produced and released after cell death by cyanobacteria and are a product of their secondary metabolism and thus, are not essential for their growth or development. These toxic molecules also designated as compounds have been described as being associated with great biological damage in several life forms including humans such as hepatotoxicity (microcystins, nodularins), neurotoxicity (anatoxins, saxitoxins), and cytotoxicity (cylindrospermopsins) and have also been recognized for their genotoxic (microcystins, cylindrospermopsins) and carcinogenic potential (microcystins, cylindrospermopsins) [1]. Cases of human mortality and morbidity have been attributed to cyanobacteria poisoning, such as the death of 60 patients in a dialysis center in Caruaru (Brazil) where the presence of microcystins in the water was the main cause and the intoxication of 148 people after a bloom of *Cylindrospermopsis raciborskii* cylindropermopsins producer in a reservoir in Queensland (Australia) [2,3,4]. Cyanotoxins are, therefore, important to surveil for human safety. Anthropogenic pressures generally cause ecosystems to become eutrophic as this leads to the intensification of cyanobacteria and respective cyanotoxicity, reducing the capability of use of those ecosystems for the several demands they currently carry, namely socio-economic activities such as water provision, irrigation, or even tourism. In cyanotoxins evaluation, several methods can be applied, namely the analytical through High pressure liquid chromatography (HPLC), Liquid chromatography mass spectrometry (LC/MS), or even ELISA (Enzyme-Linked Immunosorbent Assay) that altogether have the main advantage of enumerating cyanotoxins, allowing one to infer the toxicity level of a given sample or ecosystem by comparing the obtained value with the adopted guideline. Other methodologies applied in cyanotoxins identification are the molecular methods either through simple PCR, quantitative PCR, or even through DNA sequencing of amplified genes (structural or toxicity). Overall, these methods permit the characterization of the cyanotoxicicty that is present in a given environment either if it is free or contaminated by a bloom event. In both cases, these methods are important tools to obtain a fast assessment, identification, and characterization of the presence of the genes associated with cyanotoxins occurrence. With this, it may be possible to establish the potential hazards and risks to humans and wildlife that are associated with these freshwater ecosystems since bioaccumulation and biomagnification have been reported [5,6]. Although less labor intensive than the analytical ones, these methods have been commonly applied in cyanobacteria research in a great variety of matrices such as water, pure cultures, or soil. The research advancement of these methods currently allows the prompt identification of genes associated with microcystins (*mcyA*), cylindrospermopsins (*cyrABCJ*), anatoxin-a (*anaC*), and saxitoxins (*sxtAGI*) [7,8,9,10,11,12,13]. Other tools allow the identification of *Microcystis* microcystins-producing genus (*mcyBCDEFG*) and *Dolichospermum* anatoxin-a-producer [12,14,15].

In Portugal, almost all of the main cyanotoxins have been identified. In fact, there is a recent report (1993) of a possibly linked cyanobacteria intoxication that resulted in the loss of 20 patients in a dialysis unit in a Hospital in the South Region, however no cyanotoxins were measured [16,17]. However, it was not until the end of the 1980s that microcystins were firstly identified and characterized, becoming the first cyanotoxin to be identified in Portuguese freshwater ecosystems [18]. In this study, *Microcystis aeruginosa* was also found to be the main bloom-forming species and microcystins-producing cyanobacterium in Portugal. Thereafter, Pereira et al. [19] identified the presence of the neurotoxin saxitoxins in a reservoir in the South Region of Portugal (Montargil Reservoir). In this study, an isolated strain of *Aphanizomenon flos-aquae* was found to be the main saxitoxins producer. Recently, Oswald et al. [20] described the presence of anatoxin-a producing strains in Portugal, but these were only able to produce the cyanotoxin after laboratory cultivation, failing, until now, the environmental detection of anatoxin-a in Portugal. In all these studies, chemical methods through HPLC detection were the methods that were mainly employed. Investigations on Portuguese freshwater systems were afterwards characterized through microcystins occurrence, and it was not until 2012 that the presence of the cytotoxin cylindrospermopsins in a lagoon in the Centre Region (Vela Lagoon) [21] was established. In this study, the authors established, with the aid of DNA sequencing, a positive amplicon of a cylindrospermopsin *cyrC* gene that belonged to *Chysosporum* genus. This became the main producer of this cyanotoxin in Portugal, which is similar to what has been observed for this cyanotoxin in other European countries. The particularity of this study in comparison with the previous cyanotoxins reports in Portugal was the multi-method approach that was applied, meaning that so far, this is the only study to use molecular methods in combination with analytical methodologies (HPLC; LC/MS and ELISA) in the characterization of the reported cyanotoxin. Although chemical methods are more laborious to apply in a cyanobacterial monitoring approach, a molecular and immunological survey was conducted in seven Portuguese freshwater systems located in the North and Center Regions of Portugal. The samples were assessed for the presence of microcystins, cylindrospermopsins, anatoxin-a and saxitoxins. The molecular methods allowed the evaluation of the presence of these in water samples obtained from bloom and non-bloom events and compared these with the results from the ELISA immunoassays. Results reveal the presence of genes associated with the anatoxin-a a and saxitoxins in the North and Center Regions of the country. Cylindrospermopsins were reported in other locations besides Vela Lagoon through molecular analysis. Overall, results demonstrate the recent multiplication of cyanotoxins in national territory and an increase in risk exposure to the national population since most of the ecosystems studied are used for drinking, irrigation, and recreational purposes. Our study demands the urgent implementation of cyanotoxins programs besides the legislated MC-LR in the whole national territory in order to improve the safety of citizens, improve water quality, and water management.

## 2. Results

### 2.1. Bloom Frequency and Composition

Blooms were detected in several of the sampling sites with an overall frequency of 24% during the sampling season. Sampling sites affected by blooms comprise Vela Lagoon (June), River Tâmega (July, August, September), Torrão Reservoir (August, September), and Porto City Park Lake 2 (September), corresponding to a total of 57% of the sampling sites with bloom occurrences during the six-month sampling period. Bloom composition comprises either solely of *Microcystis* strains or a mixture of these with other filamentous strains belonging to the genera *Dolichospermum* and *Chrysosphorum*.

### 2.2. Microcystins

**Figure 1:**
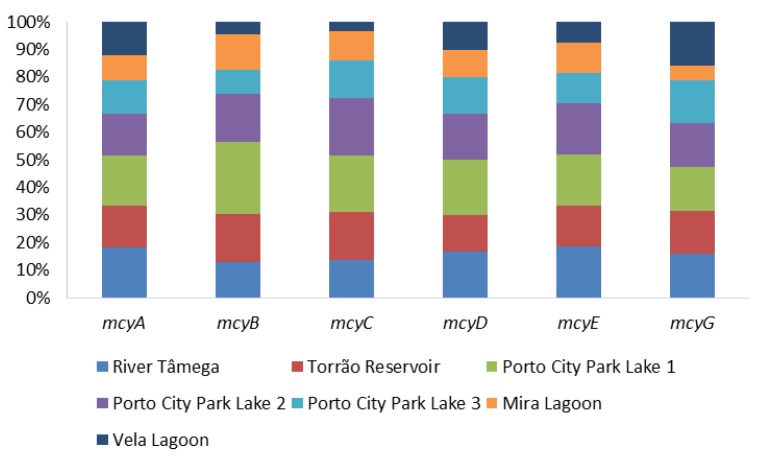
Proportion of microcystins genes (mcyABCDEG) per sampling site.

**Figure 2:**
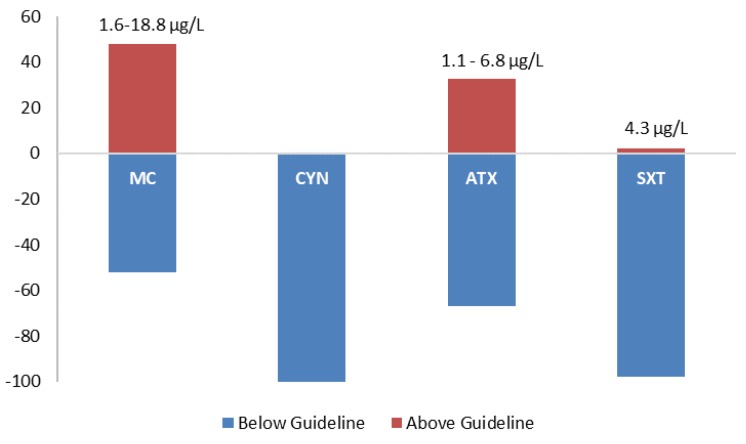
ELISA proportion values obtained in the samples in comparison with the proposed guideline value for each of the cyanotoxins tested (microcystins 1 μg/L; cylindrospermopsins 1 μg/L; anatoxin-a 1 μg/L; and saxitoxins 3 μg/L).

The presence of microcystins was evaluated through the PCR amplification of the general genetic marker mcyA and the Microcystis microcystins-producing (mcyBCDEG) and the ELISA Plate kit for Microcystin-ADDA in water samples. Results from the molecular amplification of each microcystin marker are shown in Figure 1 and Table 1. In mcyA River Tâmega and Porto City Park, Lake 1 had the highest detection, with 100% (all months) of the samples testing positive. Torrão Reservoir and Porto City Park Lake 2 had the second highest score for this gene, with both showing 83.3% (all months except May) of detection in all samples. Porto City Park Lake 3, Mira Lagoon, and Vela Lagoon had the lowest detection with 66.7% (all months except May and June), 50% (July, August, and September), and 66.7% (all months except April and June), respectively. mcyB showed only one sampling site with 100% positives and this belonged to Porto City Park Lake 1. River Tâmega, Torrão Reservoir, Porto City Park Lake 2, and Mira Lagoon had the respective percentages of 50% (July, August, and September), 66.7% (all months except May and June), 66.7% (all months except April and July), and 50% (July, August, and September). The lowest values detected of mcyB belonged to the samples of the sampling sites Porto City Park Lake 3 and Vela lagoon, with the respective percentages of 33.3% (August and September) and 16.7% (July). Overall, mcyC had higher percentages of detection in comparison to mcyB, with two sampling sites, Porto City Park Lake 1 and Lake 2, with 100% of positives (all months). The lower values of detection belonged to River Tâmega, Torrão Reservoir, Porto City Park Lake 3, and Mira Lagoon, with the respective percentages of 66.7% (all months except April and May), 83.3% (all months except May), 66.7% (all months except April and May), and 50% (July, August, and September). The lowest percentage of detection for this gene belonged to the sampling site Vela Lagoon, with a total of 16.7% of positives (July). In mcyD Porto City Park, Lake 1 was the only sampling site with all sampling months testing positive (100%). River Tâmega and Porto City Park Lake 2 had a lower percentage of detection of 83.3% in both sampling sites in distinct sampling months of the River Tâmega in all except the month of June and Porto City Park Lake 2 in all except the month of April. Torrão Reservoir and Porto City Park Lake 3 each had 66.7% of positives, both in all except the months of May and June. The lowest percentage corresponded to the two sampling sites located in the Centre Region (Mira Lagoon and Vela Lagoon), both with 50% of the samples testing positive, and these belonged in both sampling sites to the months of July, August, and September. In mcyE, none of the sampling sites obtained a 100% percentage of detection. Nonetheless, the highest value belonged to River Tâmega, Porto City Park Lake 1 and Lake 2, all with 83.3%, except in the month of April. Lower values were obtained for this genetic marker and those belonged to the sampling sites Torrão Reservoir, Porto City Park Lake 3, and Mira Lagoon, with the first with 66.7% (all except April and May) and the other two with 50% in July, August, and September. The lowest value belonged to Vela Lagoon with a total percentage of 33.3% (July and August). Finally, in mcyG, the results in all sampling sites were the lowest in comparison with the other tested markers. In this sense, the highest percentage belonged to all the sampling sites excluding Mira Lagoon, which in total obtained 50% of positives (July, August, and September), with the latter having only 16.7% (September). Regarding ELISA data for microcystins, all samples tested positive for this cyanotoxin in all sampling sites. When being compared to the established guideline by the national law decree and the WHO of 1 μg/L, 48% of the samples were above (1.6–18.8 μg/L) this guideline value and these were found in all sampling sites in at least one of the sampling months (Table 1). The only sampling site that had values above 1 μg/L in all the months was Porto City Park Lake 1 (North region). Values below the adopted guideline corresponded to a total of 52% and these occurred in all sampling months of all sampling sites besides Porto City Park Lake 1 (Figure 2).

### 2.3. Cylindrospermopsins

**Figure 3:**
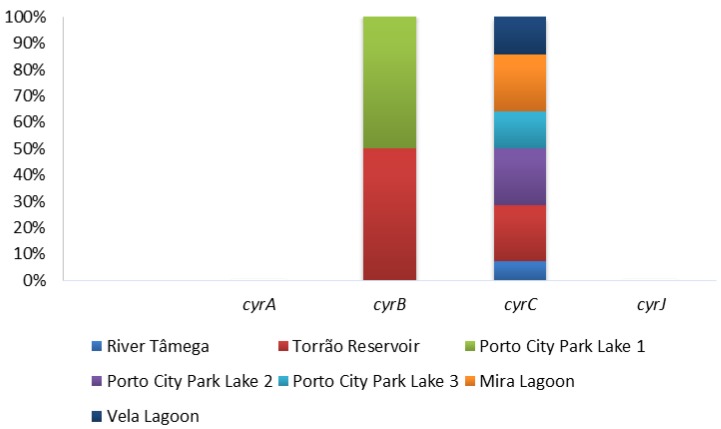
Proportion of cylindrospermopsins genes (cyrABCJ) per sampling site.

The presence of cylindrospermopsins was evaluated through PCR amplification of four general primers (cyrABCJ) in all water samples and the ELISA Plate kit for cylindrospermopsins only in Vela Lagoon was the first site to report the presence of cylindrospermopsins in Portugal. Of all the four genetic markers tested, only two gave positive results and these include the cyrB and cyrC (Figure 3; Table 1). Both cyrA and cyrJ were negative in all the sampled months in all the sampling sites (Figure 3; Table 1). In cyrB, only two sampling sites gave positive results for this gene, Torrão Reservoir and Porto City Park Lake 1. Both had a total percentage of 16.7% in distinct months—namely, September in Torrão Reservoir and June in Porto City Park Lake 1. In the remaining sampling sites, all tested negative for this genetic marker. In cyrC, all sampling sites had at least one of the samples testing positive with the exception of Porto City Park Lake 1, where no positives were found to occur in any of the sampled months. The highest percentage of detection corresponded to the sampling sites Torrão Reservoir, Porto City Park Lake 2, and Mira Lagoon, all with 50% of positives. In these distinct sampling months, they tested positive and these include Torrão Reservoir in April, June, and July; Porto City Park Lake 2 in July, August, and September; and Mira Lagoon in June, July, and September (Table 1). A lower percentage belongs to the sampling sites Porto City Park Lake 3 and Vela Lagoon, both with 33.3% of positives. In these, Porto City Park Lake 3 had positives in the months of August and September, while in Vela Lagoon, these occurred in the months of June and July (Table 1). The lowest value corresponded to River Tâmega, with 16.7% of positives and this corresponded to the month of June. In the ELISA immunoassay, all samples of Vela Lagoon gave positive detection excluding those belonging to the months of July and September, which are all below the proposed guideline value of 1 μg/L (Table 1; Figure 2).

### 2.4. Anatoxin-a

**Figure 4:**
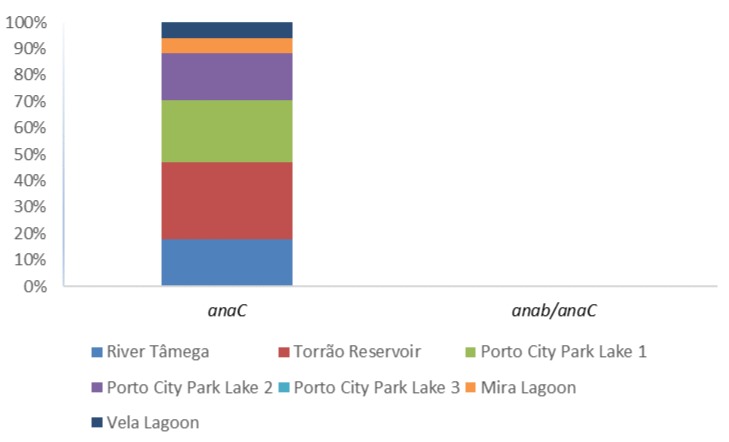
Proportion of anatoxin-a gene (anaC) and Dolichospermum sp. anatoxin-a producer (anab/anaC) per sampling site.

The presence of anatoxin-a was evaluated through the PCR amplification of the general marker anaC gene and the specific Dolichospermum-anaC gene (Table 1; Figure 4). Also, enumeration in water samples was conducted through ELISA Plate Anatoxin—a kit in all 42 water samples (Table 1). In the PCR amplification, only Porto City Park Lake 3 showed negative results in all the tested months. Nonetheless, other positives were obtained and these include, in a higher percentage, the sampling site Torrão Reservoir in all except the month of June. Porto City Park Lake 1 had the following high percentage, which corresponded to 66.7%, belonging to all the sampling months except April and July. Porto City Park Lake 2 and River Tâmega all had a 50% detection and this corresponded in both sampling sites to the months of July, August, and September. The specific Dolichospermum-anaC gene marker was also tested in all 42 water samples and a positive amplicon was observed in neither. Regarding ELISA Plate assay, 33% of the samples gave above (1.1–6.8 μg/L) the guideline value for this cyanotoxin (1 μg/L) (Table 1; Figure 2). In these, Porto City Park Lake 1 had all but one of the months (July) with values above this guideline. Other sampling sites that had at least one of the months above 1 μg/L of anatoxin-a include Porto City Park Lakes 2 and 3, Mira, and Vela Lagoons.

### 2.5. Saxitoxins

**Figure 5:**
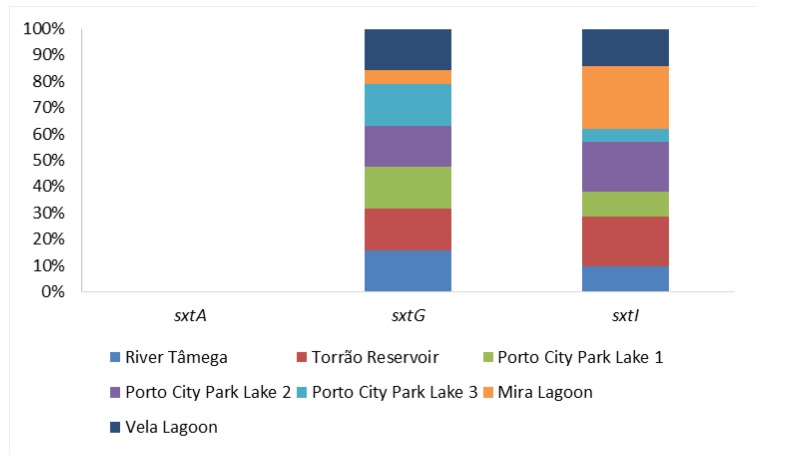
Proportion of saxitoxins genes (sxtAGI) per sampling site.

The presence of saxitoxins were evaluated through the PCR amplifications of three distinct genetic markers belonging to the sxt gene cluster (sxtAGI) (Table 1; Figure 5) and the ELISA Plate Kit for saxitoxins (Table 1). Regarding PCR amplifications of sxtA, none of the samples gave a positive result for this genetic marker (Figure 5). In sxtG, all sampling sites gave 50% positives except Mira Lagoon, where the percentage of detection corresponded to 16.7%. All the 50% of detection had positives in the sampling months of July, August, and September. In Mira Lagoon, the only positive belonged to the month of September. In sxtI, the highest percentage of detection was in the sampling site Mira Lagoon, with 83.3% of positives in all except the month of April. The second highest percentage of detection belonged to the sampling sites Torrão Reservoir and Porto City Park Lake 2, with 66.7% of positives in all except the month of June as well as in May in Torrão Reservoir and April in Porto City Park Lake 2. A lower percentage of detection belonged to Vela Lagoon, with 50% of positives in all except the months of April, July, and September. The lowest percentage of detection belonged to the sampling sites River Tâmega and Porto City Park Lake 1, both with 33.3% of positives, and to Porto City Park Lake 3, with 16.7% of positives. In River Tâmega, positives were obtained in all but the months of April, June, May, and September, while in Porto City Park Lake 1, positives were obtained in all except the months of April, May, June, and July. In the Porto City Park Lake 3 sampling site, the only positive belonged to the month of September. Regarding the ELISA Plate Kit assays, this resulted in only one sample belonging to Porto City Park Lake 2 from the month of August which had a value above (4.3 μg/L) the guideline value (3 μg/L) (Table 1; Figure 2). All the remaining 41 water samples were below the proposed guideline value.

### 2.6. DNA Sequencing

All the positive amplicons belonging to the biosynthetic gene clusters of anatoxin-a and saxitoxins were purified and sequenced. All the fragments gave above 97% of identity through the BLAST search tool to Chrysosporum sp. strains in the anaC, sxtG, and sxtI amplicons.

## 3. Discussion

This study analyzed seven freshwater systems located in two regions of Portugal (North and Center) for the presence of the four main cyanotoxins (microcystins, cylindrospermopsins, anatoxin-a, and saxitoxins) through the application of a two-method approach in its surveillance. In this study, both molecular (PCR and DNA sequencing) and ELISA assays were employed in a combined manner to first detect and identify the presence of these cyanotoxins (molecular methods) and second to enumerate the toxins directly from water samples (ELISA assays). Previous data in Portugal revealed that *Microcystis aeruginosa* is the main bloom-forming species [18]. However, in our study, a shift in bloom composition was observed, with this corresponding to an exceptionally warm year with two heat waves occurring between May and October which resulted in intense wildfires and the loss of more than 60 people. The occurrence of these heat waves is well supported by the fact that more than 50% of the ecosystems studied presented blooms in the sampling dates. This confirms that in Portugal, bloom occurrence may be becoming intrinsically associated with climate change events, although further surveillance will resolve this question. In the bloom samples, the composition belonged to either *Microcystis* alone or a mixture of *Microcystis*, with two other filamentous genera belonging to *Dolichospermum* and *Chrysosporum*, a phenomenon that has not been previously observed in Portuguese freshwater systems, particularly in the sampled regions where *Microcystis* was, until this study, the only bloom-forming genus [18]. Freshwater ecosystems carry with them several impacts on drinking, irrigation, and recreation and several socio-economic activities have been attributed such as agriculture, sports, or even tourism. All carry with them several contact routes of exposure, particularly if blooms are present since the release of cyanotoxins in high amounts is also a well-known feature. Therefore, dermal, inhalation, or ingestion of contaminated water either directly or indirectly can occur if populations are misinformed, although the lack of epidemiologic data on cyanotoxins contaminations in Portugal does not allow us to infer if any citizen or animal was affected by these blooms or cyanotoxins in the year 2017 in any of the sampled regions or ecosystems.

Regarding the surveillance for cyanotoxins, previous studies reveal that microcystins are well distributed while the saxitoxins were circumscribed to the South region and only recently in 2012 were cylindrospermopsins found to occur in Portuguese freshwater systems, however only a Center Region lagoon was monitored (Vela Lagoon) [21]. In our study, the number of ecosystems analyzed increased and revealed the overall expansion or the multiplication of all the main cyanotoxins with particular emphasis to the cylindrospermopsins (Center Region to North Region) through molecular analysis and saxitoxins (South Region to North and Center regions) by both molecular and ELISA immunoassays. In regard to the presence of anatoxins-a, it was observed in a new report in Portugal either through the PCR amplification of *anaC* gene or by measuring the cyanotoxin itself through the respective ELISA immunoassay. This cyanotoxin was enumerated in distinct amounts in all the sampled sites, however since they are considered potent toxins, the guideline value proposed for a cyanotoxins risk assessment in Portuguese freshwater systems was 1 µg/L, similarly to microcystins and cylindrospermopsins.

Regarding molecular methods, results showed that for microcystins, with all the gene markers applied in this study, in the North Region, they are more prevalent than in the Center Region where most of the genes were only positive in only one of the sampled months. Measurement of microcystins showed that this cyanotoxin was present in all the ecosystems studied, in all the months, and in all the 42 samples that were analyzed. The registered values for this cyanotoxin were distinct and overall gave less but close to 50% of the samples above the proposed guideline, which is 1 µg/L. Values ranged between 1.6 to 18.8 µg/L and were detected under bloom scenarios such as those that occurred in River Tâmega and Torrão Reservoir in August and September, Porto City Park Lake 1 in July, and Porto City Park Lake 2 in September. Cylindrospermopsins were, as previously referred, only detected in Vela Lagoon in 2012, but in the following year, they were not found by any of the methods employed [21]. For this reason, the absence of cylindrospermopsins was only measured again in our experiments in Vela Lagoon through the respective ELISA immunoassay, while through molecular analysis, it was analyzed in all the seven ecosystems, again including Vela Lagoon. Results show the spread of cylindrospermopsins genes apart from the initially described Vela Lagoon, particularly to the North Region ecosystems and also to the other Center Region ecosystem analyzed (Mira Lagoon). The lack of confirmation through DNA sequencing is due to the fact that previously, the cylindrospermopsins-producing genus has been identified as belonging to *Chrysosporum* sp., similarly to other European countries not expecting the emergence of other producing genus in European freshwaters, which also includes Portugal [21,22]. Of the four genetic markers applied in cylindrospermopsins surveillance, only two gave positive results in all of the samples, while *cyrA* and *cyrJ* were negative, failing their amplification and presence in Portuguese freshwater systems. However, *cyrC* gave the highest percentage of detection in all ecosystems excluding the sampling site Porto City Park Lake 3 where none of the samples were positive. The data from this study shows that *cyrC* is still the best candidate marker of cylindrospermopsins in Portugal apart from the well described *cyrJ* [11,23,24]. Neurotoxins were also analyzed for their presence and enumeration in all the water samples collected in this study. Anatoxin-a, one of the neurotoxins surveilled, revealed its first detection by molecular methods and ELISA Plate Kit immunoassays. DNA sequencing further revealed that all the positive amplicons belonged to the genus *Chrysosporum* sp., a result that is further confirmed by the absence of positives in the *Dolichospermum* sp. specific anatoxin-a PCR experiments. In this sense, the main producing genus of anatoxin-a in Portuguese freshwater systems seems to belong to the genus *Chrysosporum* in contrast to *Dolichospermum*. PCR results also highlighted the high prevalence of anatoxin-a genes in Portugal, in particular in the North Region ecosystems, which is in contrast to the Center Region ecosystems where only one of the samples tested positive for this cyanotoxin. Saxitoxins were also analyzed and in molecular methods, three distinct genetic markers were applied, all belonging to the *sxt* gene cluster. Of the three tested markers, only *sxtG* and *sxtI* gave positive amplifications, while *sxtA* was negative in all of the samples. Although they are all involved in the amplification of the *sxt* gene cluster, it seems that *sxtG* and *sxtI* are good candidate markers for saxitoxin presence in Portuguese freshwater systems rather than *sxtA*. The lack of amplification of *sxtA* could be due to the absence in the filtered biomass of this gene or also due to the lack of the saxitoxin cyanobacterium producer in the same sample. Previous environmental analysis in Polish lakes applying these three genetic markers showed that sxtA can be negative in environmental samples [25]. Authors from this study suggested that the lack of amplifications could be due to either sequence dissimilarities between targets and primers, although sxtA is based in *Chrysosporum* sp. DNA sequences, or due to mutations (insertions and deletions) in the sxt gene cluster which would result in a negative amplification [25]. Since Portuguese samples were all negative for sxtA, we suggest that these negative results could be mainly attributed to insertions and deletions in the DNA sequences of the sxt gene cluster of North and Center Chrysosporum sp. since the reference strain Chrysosporum gracile LMCYA 040 is a South region isolate. Nonetheless, in further national surveillance campaigns, the *sxtA* gene marker should be included. DNA sequencing further revealed that all the positive amplicons belonged to the genus *Chrysosporum* sp., denoting that strains belonging to this genus are responsible for neurotoxin production in Portuguese freshwater systems, particularly in the North and Center Regions ecosystems. These results are in agreement with bloom observation, highlighting that climate change events may be favoring the growth of neurotoxin producing strains in Portugal. Regarding ELISA data, the results show that only one of the total 42 samples analyzed were above the guidelines proposed for this cyanotoxin and this occurred in a North Region ecosystem.

The percentage of correlation between positive amplicons and positive cyanotoxins enumerations were evaluated. In regard to microcystins, the correlation was 78%, and both neurotoxins had values below 50% of positive correlations between the two techniques, while in cylindrospermopsins, only one sample was positive by the two methods. However, this was the first study to conduct this analysis in all cyanotoxins. The elevated number in microcystins shows that for this cyanotoxin and in Portuguese freshwater systems, the application of a molecular surveillance is well correlated with the presence of the cyanotoxin itself. However, lower values were obtained in the other three cyanotoxins, revealing the need to incorporate other methodologies besides the molecular method. Although ELISA immunoassays can enumerate congeners that closely resemble cyanotoxins molecules, overall, the application of both techniques can become a first approach in a cyanotoxicity scenario of a given ecosystem, allowing one to redirect the chemical assays for cyanotoxin enumeration since in these, the screening of all the cyanotoxins is labor intensive and so far only three of them have been proposed to be enumerated simultaneously [26]. As all of the studied ecosystems had cyanotoxins detected by the two methods and since they are considered mainly eutrophic, this leads to the need for constant surveillance and redirecting further analysis of cyanotoxins in the studied ecosystems. Also, if climate change events become frequent in Portugal, similarly to other European countries, the application of molecular and ELISA immunoassays can still provide us with epidemiologic data on cyanotoxins occurrence in Portuguese freshwater systems.

The multiplication of two of the four main cyanotoxins studied in Northern and Central regions and the first environmental appearance of anatoxin-a in Portugal reveals high concern regarding the elevated risks that the national population is facing, which requires urgent surveillance, namely through chemical analysis (LC/MS) to confirm some of the positive results that were obtained in this study. The lack of confirmation in our samples is due to the fact that LC/MS is an expensive and high expertise technique while PCR and ELISA can serve as directional techniques in the cyanotoxicity evaluation prior to any chemical survey. In this sense, deeper investigations should be carried out in order to properly assess cyanotoxins occurrence in Portuguese freshwater systems, namely through chemical surveys (LC/MS). This will provide us with epidemiological data to prevent future cyanotoxins episodes such as the one that occurred in a dialysis unit in a Southern Hospital in 1993 in Portugal. Investigations are therefore important to provide data besides the legislated microcystins-LR in Portuguese freshwater systems.

## 4. Conclusions

The surveillance of four main cyanotoxins (microcystins, cylindrospermopsins, anatoxin-a, and saxitoxins) in two Regions of Portugal highlighted, via a two-method approach, the multiplication of cyanotoxins that were initially circumscribed to the Center (cylindrospermopsins) and South (cylindrospermopsins and saxitoxins) to the North and Center regions apart from the well distributed microcystins. Anatoxin-a was firstly detected in the sampled ecosystems by the two methods approach, which required further investigations, namely through chemical methods (LC/MS), to determine the effective presence of this cyanotoxin in Portuguese freshwater systems. With this study, it becomes possible to improve the risk assessment strategy for cyanotoxins in Portugal through the screening of multiple cyanotoxins apart from the legislated microcystins–LR.

## 5. Materials and Methods

### 5.1. Sampling

A total of seven freshwater ecosystems located in the North and Centre Regions of Portugal were surveyed between April and September of 2017, encompassing a total of 42 samples (Table 2). Water samples were collected from the near shore of each sampled ecosystem and when blooms were present, water was collected from the surrounding bloom biomass. Presence of blooms was registered and biomass was collected with a plankton net to determine bloom composition. Further, 15 mL of water was also collected for ELISA immunoassays. All samples were brought to the laboratory under refrigerated conditions. Water samples for the molecular analysis were immediately processed upon arrival. These were filtered with a vacuum filtration system using glass-microfiber filters (grade MG C, 47 mm diameter, 1.2 μm porosity) (Munktell^®^, Falun, Sweden). The filtered biomass was maintained at −20 °C until further DNA extraction. Water samples for ELISA immunoassays were frozen at −20 °C prior to their analysis.

### 5.2. DNA Extraction

DNA was extracted by scraping the filtered biomass with a sterile scalpel and the removed biomass was placed in a sterile Eppendorf tube. We then proceeded to DNA extraction using the PureLink™ Genomic DNA Kit (Invitrogen, Carlsbad, CA, USA) following the protocol for Gram-negative bacteria. DNA was eluted in 50 μL of elution buffer. Genomic DNA was stored at −20 °C until further PCR amplifications.

### 5.3. PCR Amplifications

All PCR reactions were performed using the GoTaq^®^ DNA polymerase (Promega, Madison, WI, USA) in a volume of 20 μL containing 1 × PCR buffer, 2.5 mM MgCl_2_, 250 μM of each deoxynucleotide triphosphate, 10 pmol of each primer, and 0.5 U of Taq DNA polymerase. Microcystins *mcyA* PCR amplification was performed using the primer pair mcya-cd1F/mcya-cd1R with the following PCR conditions: an initial denaturation at 95 °C for 2 min and 35 cycles at 95 °C for 90 s, 56 °C for 30 s, and 72 °C for 50 s (Table 3) [8]. Microcystins of *Microcystis*-*mcyBCDEG* specific PCR amplifications were carried out using the primer pairs 2156F/3111R (*mcyB*), PSCF1/PSCR1 (*mcyC*), PKDF1/PKDR1 (*mcyD*), PKEF1/PKER1 (*mcyE*), and PKGF1/PKGR1 (*mcyG*) with the following conditions: an initial denaturation at 94 °C for 5 min and 35 cycles at 95 °C for 60s, 52 °C for 30 s, and 72 °C for 60s (Table 3) [14,15]. A *Microcystis aeruginosa* microcystins-producing strain LEGE 00063 was used as a positive control in all the microcystins PCR experiments. Cylindrospermopsins PCR amplification was carried out using the primer pairs AMTFw/AMT Rev (*cyrA*), M13/M14 (*cyrB*), K18/M4 (*cyrC*), and cynsulfF/cylnamR (*cyrJ*) (Table 3). In *cyrA*, the PCR conditions were as follows: an initial denaturation at 94 °C for 3 min and 30 cycles at 94 °C for 10 s, 50 °C for 20 s, and 72 °C for 60 s [9]. In *cyrB* and *cyrC*, the PCR conditions were as follows: an initial denaturation at 94 °C for 10 min and 30 cycles at 94 °C for 30 s, 45 °C for 30 s, and 72 °C for 60 s [7]. In *cyrJ*, the PCR conditions were as follows: an initial denaturation at 94 °C for 3 min and 30 cycles at 94 °C for 10 s, 57 °C for 20 s, and 72 °C for 60 s [11]. A *Cylindrospermopsis raciborskii* cylindrospermopsin-producing strain LEGE 97047 was used as a positive control in all the cylindrospermopsins PCR experiments. Anatoxin-a PCR amplification was carried out using the primer pairs anaC-genF/anaC-genR (anaC) and anaC-anabF/anaC-anabR (*Dolichospermum*-*anaC* specific), both with the following conditions: an initial denaturation at 94 °C for 2 min and 35 cycles at 94 °C for 30 s, 58 °C for 30 s, and 72 °C for 30 s (Table 3) [12]. A *Dolichospermum* sp. anatoxin-a-producing strain LEGE X-002 was used as a positive control in both anatoxin-a PCR experiments. Saxitoxins through *stxAGI* genetic markers were amplified using the primer pairs sxtA855_F/sxtaA1480_R (*sxtA*), sxtG432_F/sxtG928_R (*sxtG*), and sxtI682F/sxtI877R (*sxtI*) (Table 3). In *sxtA* and *sxtG*, the PCR conditions were as follows: an initial denaturation at 98 °C for 30 s and 35 cycles at 98 °C for 5 s, 62 °C for 5 s, and 72 °C for 10 s [13]. In *sxtI*, the PCR conditions were as follows: 94 °C for 3 min and 35 cycles at 94 °C for 10 s, 52 °C for 20 s, and 72 °C for 60 s [10]. A *Chrysosporum gracile* strain LMECYA 040 was used as a positive control in all the saxitoxins PCR experiments. The final extension in all PCR amplifications was performed at 72 °C between 5 to 7 min. All PCR amplifications were carried out in a Veriti thermal cycler (Applied Biosystems, Foster City, CA, USA).

### 5.4. DNA Sequencing

100 µl of PCR reaction of each amplified product belonging to neurotoxin biosynthetic genes (anatoxin-a and saxitoxins) was purified using the PureLink™ Quick Gel Extraction and PCR Purification Combo Kit (Invitrogen, Carlsbad, CA, USA) in accordance with the manufacturer protocol followed by direct sequencing (Macrogen, Madrid Spain). The obtained nucleotide sequences were initially examined using BLAST and the NCBI nucleotide database (http://www.ncbi.nlm.nih.gov/BLAST).

### 5.5. ELISA Immunological Assays

Intracellular and extracellular concentrations of microcystins, cylindrospermopsins, anatoxin-a, and saxitoxins were measured using the commercially available ELISA Plate kits (Abraxis Inc., Warminster, PA, USA). All 15 mL water samples were initially filtered with 0.2 µm filters to remove intact cyanobacteria cells following frosting at −20 °C. Then, the samples were subjected to three freeze-and-thaw cycles to lyse the intact cells and release the intracellular toxins. Triplicates for each sample were performed and the procedure followed the manufacturer’s protocol accordingly. Total cyanotoxin absorbance was measured in a plate reader (BioTek Instruments, Inc., Winooski, VT, USA) at 450 nm. All 42 water samples were measured for microcystins, saxitoxins, and anatoxin-a, while only the six samples belonging to Vela Lagoon were subjected to cylindrospermopsins ELISA Plate Kit.

## Figures and Tables

**Table 1:** Summary of the PCR and ELISA results for each cyanotoxin measured per sampling site.

Cyanotoxins	MC							CYN					ATX			SXT			
Assessment	PCR						ELISA (µg/L)	PCR				ELISA (µg/L)	PCR		ELISA (µg/L)	PCR			ELISA (µg/L)
Location	*mcyA*	*mcyB*	*mcyC*	*mcyD*	*mcyE*	*mcyG*		*cyrA*	*cyrB*	*cyrC*	*cyrJ*		*anaC*	*anab/anac*		*sxtA*	*sxtG*	*sxtI*	
**River Tâmega**																			
April	+	-	-	+	-	-	0.3	-	-	-	-	ND	-	-	0.2	-	-	-	0.0
May	+	-	-	+	+	-	0.2	-	-	-	-	ND	-	-	0.7	-	-	-	0.1
June	+	-	+	-	+	-	0.5	-	-	+	-	ND	-	-	0.2	-	-	-	0.1
July	+	+	+	+	+	+	0.4	-	-	-	-	ND	+	-	0.3	-	+	+	0.1
August	+	+	+	+	+	+	18.8	-	-	-	-	ND	+	-	1.0	-	+	+	1.1
September	+	+	+	+	+	+	17.0	-	-	-	-	ND	+	-	0.8	-	+	-	0.1
**Torrão Reservoir**																			
April	+	+	+	+	-	-	2.7	-	-	+	-	ND	+	-	0.3	-	-	+	0.1
May	-	-	-	-	-	-	0.3	-	-	-	-	ND	+	-	1.0	-	-	-	0.1
June	+	-	+	-	+	-	0.3	-	-	+	-	ND	-	-	0.4	-	-	-	0.1
July	+	+	+	+	+	+	0.2	-	-	+	-	ND	+	-	0.2	-	+	+	0.3
August	+	+	+	+	+	+	16.0	-	-	-	-	ND	+	-	0.3	-	+	+	0.7
September	+	+	+	+	+	+	5.0	-	+	-	-	ND	+	-	0.2	-	+	+	0.2
**Porto City Lake 1**																			
April	+	+	+	+	-	-	4.3	-	-	-	-	ND	-	-	5.2	-	-	−	0.2
May	+	+	+	+	+	-	11.3	-	-	-	-	ND	+	-	2.1	-	-	-	0.3
June	+	+	+	+	+	-	3.5	-	+	-	-	ND	+	-	6.8	-	-	-	0.2
July	+	+	+	+	+	+	1.6	-	-	-	-	ND	-	-	0.4	-	+	-	0.1
August	+	+	+	+	+	+	2.1	-	-	-	-	ND	+	-	1.9	-	+	+	0.3
September	+	+	+	+	+	+	2.6	-	-	-	-	ND	+	-	1.6	-	+	+	0.5
**Porto City Lake 2**																			
April	-	-	+	-	-	-	0.3	-	-	-	-	ND	-	-	0.2	-	-	-	0.2
May	+	+	+	+	+	-	2.0	-	-	-	-	ND	-	-	0.4	-	-	+	0.2
June	+	+	+	+	+	-	13.3	-	-	-	-	ND	-	-	1.5	-	-	-	0.3
July	+	-	+	+	+	+	3.9	-	-	+	-	ND	+	-	0.4	-	+	+	0.9
August	+	+	+	+	+	+	4.8	-	-	+	-	ND	+	-	0.9	-	+	+	4.3
September	+	+	+	+	+	+	6.5	-	-	+	-	ND	+	-	0.6	-	+	+	2.6
**Porto City Lake 3**																			
April	+	-	-	+	-	-	0.3	-	-	-	-	ND	-	-	1.8	-	-	-	0.1
May	-	-	-	-	-	-	0.2	-	-	-	-	ND	-	-	0.4	-	-	-	0.2
June	-	-	+	-	-	-	0.1	-	-	-	-	ND	-	-	1.0	-	-	-	0.2
July	+	-	+	+	+	+	0.2	-	-	-	-	ND	-	-	1.1	-	+	-	0.2
August	+	+	+	+	+	+	0.4	-	-	+	-	ND	-	-	0.3	-	+	-	0.2
September	+	+	+	+	+	+	1.9	-	-	+	-	ND	-	-	0.4	-	+	+	0.2
**Mira Lagoon**																			
April	-	-	-	-	-	-	0.5	-	-	-	-	ND	-	-	0.4	-	-	-	0.1
May	-	-	-	-	-	-	0.3	-	-	-	-	ND	-	-	0.5	-	-	+	0.2
June	-	-	-	-	-	-	0.3	-	-	+	-	ND	-	-	0.5	-	-	+	0.6
July	+	+	+	+	+	-	0.6	-	-	+	-	ND	-	-	2.1	-	-	+	0.3
August	+	+	+	+	+	-	0.3	-	-	-	-	ND	-	-	0.6	-	-	+	0.5
September	+	+	+	+	+	+	1.9	-	-	+	-	ND	+	-	1.0	-	+	+	1.3
**Vela Lagoon**																			
April	-	-	-	-	-	-	0.3	-	-	-	-	0.1	-	-	1.6	-	-	-	1.9
May	+	-	-	-	-	-	0.3	-	-	-	-	0.1	-	-	0.5	-	-	+	1.4
June	-	-	-	-	-	-	0.4	-	-	+	-	0.1	-	-	1.7	-	-	+	1.8
July	+	+	+	+	+	+	2.1	-	-	+	-	0.0	-	-	0.9	-	+	-	1.9
August	+	-	-	+	+	+	0.9	-	-	-	-	0.1	+	-	0.8	-	+	+	1.8
September	+	-	-	+	-	+	8.7	-	-	-	-	0.0	-	-	1.1	-	+	-	2.4

(+)—positive amplification (PCR); (-)—no amplification; ND—not determined. *mcyA* amplification resulted in a positive ELISA assay in 78% of the samples; *anaC* resulted in 40%; *sxtG* in 45%; *sxtI* in 48%; *cyrB* in 0%; *cyrC* in 16.7%.

**Table 2:** Sampling sites description.

Geography	Sampling Site	Trophic Status	Ecosystem Uses	Socio-economic Activities
North Region	River Tâmega	Eutrophic	Recreational	Sports, Fishing
	Torrão Reservoir	Eutrophic	Water provision, irrigation, recreational	Agriculture, Sports, Tourism
	Porto City Park Lake 1	Eutrophic	Recreational	Tourism
	Porto City Park Lake 2	Eutrophic	Recreational	Tourism
	Porto City Park Lake 3	Mesotrophic	Recreational	Tourism
Center Region	Mira Lagoon	Eutrophic	Irrigation, Recreational	Agriculture, Sports, Tourism
	Vela Lagoon	Eutrophic	Irrigation, Recreational	Agriculture, Sports, Tourism

**Table 3:** List of primers used in this study.

Target	Primer	Primer Sequence 5′-3′	Reference
*mcy*A	mcyA-CD1F	AAAATTAAAAGCCGTATCAAA	[8]
	mcyA-CD1R	AAAAGTGTTTTATTAGCGGCTCAT	
*mcy*B	mcyB 2156-F	ATCACTTCAATCTAACGACT	[14]
	mcyB 3111-R	AGTTGCTGCTGTAAGAAA	
*mcy*C	PSCF1	GCAACATCCCAAGAGCAAAG	[15]
	PSCR1	CCGACAACATCACAAAGGC	
*mcy*D	PKDF1	GACGCTCAAATGATGAAAC	[15]
	PKDR1	GCAACCGATAAAAACTCCC	
*mcy*E	PKEF1	CGCAAACCCGATTTACAG	[15]
	PKER1	CCCCTACCATCTTCATCTTC	
*mcy*G	PKGF1	ACTCTCAAGTTATCCTCCCTC	[15]
	PKGR1	AATCGCTAAAACGCCACC	
*cyr*A	AMT Fw	ATTGTAAATAGCTGGAATGAGTGG	[9]
	AMT Rev	TTAGGGAAGTAATCTTCACAG	
*cyr*B	M13	GGCAAATTGTGATAGCCACGAGC	[7]
	M14	GATGGAACATCGCTCACTGGTG	
*cyr*C	K18	CCTCGCACATAGCCATTTGC	[7]
	M4	GAAGCTCTGGAATCCGGTAA	
*cyr*J	cynsulfF	ACTTCTCTCCTTTCCCTATC	[11]
	cylnamR	GAGTGAAAATGCGTAGAACTTG	
*ana*C	anaC-genF	TCTGGTATTCAGTCCCCTCTAT	[12]
	anaC-genR	CCCAATAGCCTGTCATCAA	
*Dolichospermum sp*. *ana*C	anaC-anabF	GCCCGATATTGAAACAAGT	[12]
	anaC-anabR	CACCCTCTGGAGATTGTTTA	
*sxt*A	sxtA855_F	GACTCGGCTTGTTGCTTCCCC	[13]
	sxtA1480_R	GCCAAACTCGCAACAGGAGAAGG	
*sxt*G	sxtG432_F	AATGGCAGATCGCAACCGCTAT	[13]
	sxtG928_R	ACATTCAACCCTGCCCATTCACT	
*sxt*I	sxtI 682F	GGATCTCAAAGAAGATGGCA	[10]
	sxtI 877R	GCCAAACGCAGTACCACTT	
